# IL-10 Induced by mTNF Crosslinking-Mediated Reverse Signaling in a Whole Blood Assay Is Predictive of Response to TNFi Therapy in Rheumatoid Arthritis

**DOI:** 10.3390/jpm12061003

**Published:** 2022-06-19

**Authors:** Marco Krasselt, Natalya Gruz, Matthias Pierer, Christoph Baerwald, Ulf Wagner

**Affiliations:** Medical Clinic III, Endocrinology, Nephrology and Rheumatology, Leipzig University, Liebigstr. 21, 04103 Leipzig, Germany; natalya.gruz@medizin.uni-leipzig.de (N.G.); matthias.pierer@medizin.uni-leipzig.de (M.P.); christoph.baerwald@medizin.uni-leipzig.de (C.B.); ulf.wagner@medizin.uni-leipzig.de (U.W.)

**Keywords:** rheumatoid arthritis, bDMARD, TNF inhibitor, IL-10, prediction, reverse signalling, whole blood

## Abstract

(1) Background: To date, the response of patients with rheumatoid arthritis (RA) to the various biologic DMARD available cannot be predicted due to a lack of reliable biomarkers. Based on our preliminary work on tmTNF reverse signaling, we developed a whole-blood assay measuring tmTNF crosslinking-induced IL-10 production to predict the response to TNF inhibitor (TNFi) therapy. (2) Methods: This prospective study included patients with active RA. Depending on the clinical judgment of the attending rheumatologist, either therapy with a TNF or JAK inhibitor was initiated. Clinical parameters and blood samples were obtained at baseline and after 8 weeks of therapy. The blood samples were collected using a newly developed whole-blood assay based on the principle of tmTNF reverse signalling. Subsequently, IL-10 was measured via enzyme-linked immunosorbent assay (ELISA) technique. (3) Results: 63 patients with RA were enrolled. In fifteen patients, TNFi therapy was initiated, while eight patients started a JAKi treatment. The cross-sectional analysis of all patients showed a positive correlation between tmTNF crosslinking-induced IL-10 and parameters of disease activity (CRP [r = 0.4091, *p* = 0.0009], DAS28 [r = 0.3303, *p* = 0.0082]) at baseline. In the TNFi treatment study, IL-10 was found to be significantly higher in EULAR responders than in non-responders (*p* = 0.0033). After initiation of JAKi treatment, in contrast, IL-10 induction was not linked to response. Longitudinal analysis of the TNFi-treated patients revealed IL-10 to decrease in responders (*p* = 0.04), but not in non-responders after 8 weeks of therapy. Of importance, the IL-10 production at baseline correlated inversely with TNFi response determined by ΔDAS28 in patients with TNFi treatment (r = −0.5299, *p* = 0.0422) while no such link was observed under JAKi therapy (*p* = 0.22). Receiver operation characteristics (ROC) analysis demonstrated a high performance of tmTNF/crosslinking-induced IL-10 in predicting a TNFi therapy response according to the EULAR criteria (AUC = 0.9286, 95% Confidence interval 0.7825–1.000, *p* = 0.0055). (4) Conclusions: In this pilot investigation, we demonstrated the feasibility of a whole-blood assay measuring tmTNF-induced IL-10 to predict clinical response to TNF inhibitor treatment. This approach might support rheumatologists in their decision for an individually tailored RA therapy.

## 1. Introduction

In today’s treatment of rheumatoid arthritis (RA), biologic disease-modifying antirheumatic drugs (bDMARDs) are indispensable. Even though conventional synthetic DMARDs such as methotrexate are recommended as the first line treatment [1], up to 70% of the patients fail to achieve remission or even low disease activity, requiring an intensified therapy [2,3]. In approx. 90% of RA patients, the first bDMARD used is a tumour necrosis factor (TNF) inhibitor [4]. Various TNF inhibitors (TNFi) are available for clinical use. All of them follow the same mechanism of action: neutralization of the inflammatory TNF cascade. To date, however, rheumatologists do not have tools to predict the response to TNFi treatment. In case of inadequate response, the chosen TNFi is often replaced by another TNFi. This so-called anti-TNF cycling is not undisputed since it possibly prolongs the time to sufficient therapy by switching to another bDMARD class with a different target [4]. Therefore, prediction of response to TNFi treatment would be of immense value for the patient, saving time and potentially preventing radiographic changes. If the rheumatologist knew whether or not an individual patient is likely to respond to TNFi therapy, he could choose the treatment option best suited for the patient (personalized medicine).

Pathogenetically, the clinical efficacy of TNFi emphasized the important role of cytokines like TNF in the pathogenesis of RA. One of the main TNF producers are monocytes, which harbour pathological subpopulations such as CD14^bright^ CD16^+^ and CD14^bright^ CD56^+^ [5,6]. In addition, monocytes of RA patients show an increased surface expression of transmembrane TNF (tmTNF) [7]. The cellular signalling pathway mediated by tmTNF ligation with soluble ligands like TNFi (reverse signalling) has been extensively investigated by us and others [7,8,9,10,11]. tmTNF crosslinking-induced reverse signalling inhibits intrinsic NFkappaB activation as well as IL-1β secretion by RA-specific monocytes and induces apoptosis [7]. Interestingly, this apoptosis is not related to TNFi response [9]. Of particular importance seems to be the tmTNF ligation-induced shedding of soluble decoy receptors such as IL-1sRI and IL-1sRII. We were able to demonstrate, that tmTNF crosslinking-induced IL-1sRII levels correlate with a good response of TNFi therapy in RA [9]. The secretion of this decoy receptor is further enhanced if tmTNF molecules are cross-linked by surface immobilized antibodies [11]. To further evaluate the value of tmTNF reverse signalling in predicting the response to TNFi therapy, we established a standardized in-vitro assay in isolated monocytes [10]. Our preliminary investigations showed that tmTNF crosslinking-induced reverse signalling led to an increased production of both, cytokine decoy receptors (sTNFR1, IL-1sRI, IL-1sRII) as well as anti-inflammatory cytokines (IL-10) in isolated monocytes and was able to predict the response to TNFi therapy [10].

The aim of the study presented was to develop and validate a standardized, whole-blood assay suitable for routine diagnostic use for the determination of tmTNF reverse signalling induced monocyte response. In a cross-sectional study with RA patients presenting consecutively to the outpatient clinic at Leipzig University, disease activity was determined and correlated with tmTNF reverse signalling induced cytokine production, regardless of the individual therapy. In 15 patients not currently treated with bDMARDs, a TNFi therapy was initiated based on current clinical guidelines, and disease activity was monitored longitudinally. The clinical goal was the prediction of TNFi response in daily practice. As a control group, RA patients initiated on a Janus kinase inhibitor (JAKi) were recruited. 

## 2. Materials and Methods

### 2.1. Human Participants

Between 2016 and 2018, a total of 63 adult patients with active RA (diagnose based on the opinion of an experienced rheumatologist and classified according to the 2010 criteria of the American College of Rheumatology (ACR) [12]) were screened for participation on this prospective open-label study. Informed consent was obtained from all participants. 48 of the patients were not initiated on TNFi therapy due to the attending rheumatologist’s clinical judgment, patient’s preference for another therapy option or relevant co-morbidities. TNFi therapy was initiated in 15 patients. Ten out of 15 patients have never been treated with bDMARDs, five patients were not currently treated with bDMARDs. Furthermore, eight patients were initiated on a treatment with a JAKi as a control group. At baseline, clinical and laboratory parameters of disease activity were obtained from all patients (*n* = 63). For the whole blood assay, blood samples were taken and cytokines were measured as described below. After initiating a therapy with a TNFi (*n* = 15) or JAKi (*n* = 8), the clinical response was evaluated (median time to evaluation: 8 weeks, 95% Confidence interval [CI] 1–4 months). Before and after therapy initiation, disease activity was obtained using the Disease Activity Score in 28 joints (DAS28-CRP). Response to therapy was judged by the EULAR response criteria [13]. Patients with any EULAR response were considered as therapy responders. The ethics committee of the University of Leipzig has approved the design of the study (Reg-No. 352/19-ek) and informed consent was obtained from all participants.

### 2.2. Whole Blood Assay

Based on our own preliminary work regarding tmTNF reverse signalling [7,8,9,10,11], we developed a kit of four pre-layered polyethylene tubes (BD Biosciences, Franklin Lakes, NJ, USA) for whole-blood sampling (4 mL of venous blood each). One “empty” control tube was used to measure the spontaneous cytokine production. A second control tube was coated with a Human IgG Fc fragment (Merck KGaA, Darmstadt, Germany). One of the test tubes was coated under sterile conditions with the TNFR2:Ig construct etanercept (Pfizer, New York, NY, USA). In a second test tube, pulverized TNFR2:Ig construct was added in addition to coating with etanercept in order to include crosslinking-independent reverse signalling. For preparation details and concentrations, see Table 1. The adhesion was conducted by incubation for 3 h at 37 °C and one cycle of washing with 1 mL phosphate buffer saline (PBS). Afterwards, the tubes were dried for 72 h at 37 °C. All tubes were subject to gas sterilization. Sodium heparin beads were added for anticoagulation (Sarstedt, Nümbrecht, Germany). Finally, the tubes were vacuumized and sealed using sterile Vacutainer^®^ caps (BD Biosciences, Franklin Lakes, NJ, USA).

### 2.3. Measurement of Cytokine Production

After collecting blood in the test tubes, they are incubated for 18 h at 37 °C and the serum is separated by centrifugation for 10 min at 3.000 rpm. The supernatant is collected and frozen at −20 °C for storage. Subsequently, IL-10 levels are measured from the supernatants by enzyme-linked immunosorbent assay ([ELISA], BD Biosciences, Franklin Lakes, NJ, USA) according to the manufacturer’s protocol.

### 2.4. Biostatistical Analysis

Continuous data were described using either mean and standard deviation (SD) or median and interquartile range (IQR). Categorical data were described by absolute or relative frequencies. To compare frequencies of categorical variables, Chi-squared tests were performed. For continuous data, Mann-Whitney U or Wilcoxon matched-pairs rank test, as appropriate, was used. Multiple comparisons after one-way ANOVA were corrected using the Holm-Sidak test. Correlation between two parameters was analysed with either Pearson’s product-moment correlation or Spearman. To estimate the power of IL-10 in predicting response, logistic regression and receiver operating characteristic (ROC) analysis were conducted. A significant statistical difference was assumed when the *p* value was less than 0.05. All analyses were conducted by using GraphPad PRISM Version 8 for Mac (GraphPad Software Inc., San Diego, CA, USA).

## 3. Results

### 3.1. Study Participants

At baseline, 63 patients with RA (mean age 61.5 years, 63.5% female) were enrolled, the majority of them being seropositive (95.2% positive for ACPA, 93.7% positive for RF). Of the patients initiated on TNFi therapy, 80% were treated with Methotrexate (in the missing patients, there was a contraindication for MTX use). Details on the individual medication are shown in Table 2.

### 3.2. Secretion of IL-10 Induced by tmTNF Crosslinking Is Linked to Disease Activity in RA Patients

**Figure 1:**
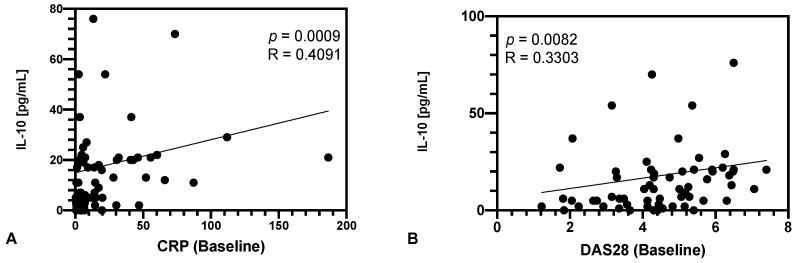
tmTNF/crosslinking-induced IL-10 at baseline in all patients (*n* = 63). Shown is the absolute IL-10 production. IL-10 is correlated to both, CRP (**A**) and DAS28 (**B**) as markers of disease activity.

The cross-sectional analysis of all patients (*n* = 63, irrespective of their subsequently initiated therapy) revealed a positive correlation between tmTNF crosslinking-induced IL-10 and parameters of disease activity (see Figure 1A for CRP [R = 0.4091, *p* = 0.0009] and Figure 1B for DAS28 [R = 0.3303, *p* = 0.0082]). No correlation with tender nor swollen joint counts could be found (data not shown).

### 3.3. tmTNF Crosslinking-Induced IL-10 at Baseline Is Increased in TNFi Responders and Decreases during Successful Therapy

**Figure 2:**
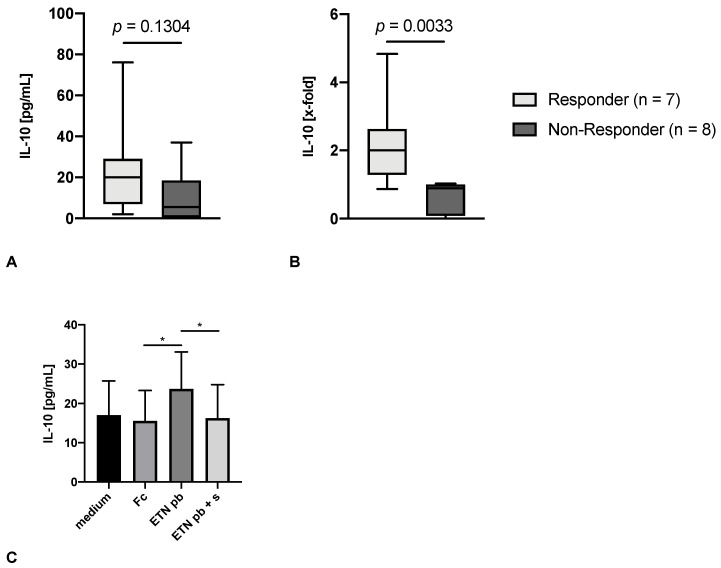
tmTNF crosslinking-induced IL-10 at baseline (*n* = 15) is higher among responders to TNFi therapy. (**A**) depicts the absolute IL-10 values while (**B**) shows the IL-10 production in relation to the human IgG Fc control (x-fold). Responders (light grey) are defined using the EULAR response criteria (any response). Depicted are boxplots and 5th to 95th percentile. (**C**) compares the mean values of absolute IL-10 levels in all test tubes of the EULAR responders (*n* = 7) using a one-way ANOVA and Holm-Sidak multiple comparison test. Depicted are the means with standard error of the mean. * *p* < 0.05.

Next, we compared the group of patients fulfilling EULAR response criteria after 8 weeks of TNFi therapy to non-responders. The results showed, that the baseline values of tmTNF crosslinking-induced IL-10 before treatment initiation were significantly higher in responders (*p* = 0.0033, Figure 2B) and were therefore predictive of the TNFi response. For the analysis, the ratio of absolute IL-10 values divided by the human IgG Fc fragment (see Table 2) was used for normalization. The absolute IL-10 values show high inter-individual variations and did not reach statistical significance (Figure 2A). To emphasize the applicability of the assay, absolute IL-10 measures from all test tubes are shown in Figure 2C.

In the subgroup under therapy with JAKi, no significant difference between responders and non-responders was seen (data not shown). After 8 weeks of TNFi therapy, tmTNF crosslinking-induced IL-10 decreased in responders (*p* = 0.0156), but not in non-responders (data not shown).

### 3.4. tmTNF Crosslinking-Induced IL-10 Is Predictive of Response to TNFi, but Not to JAKi

**Figure 3:**
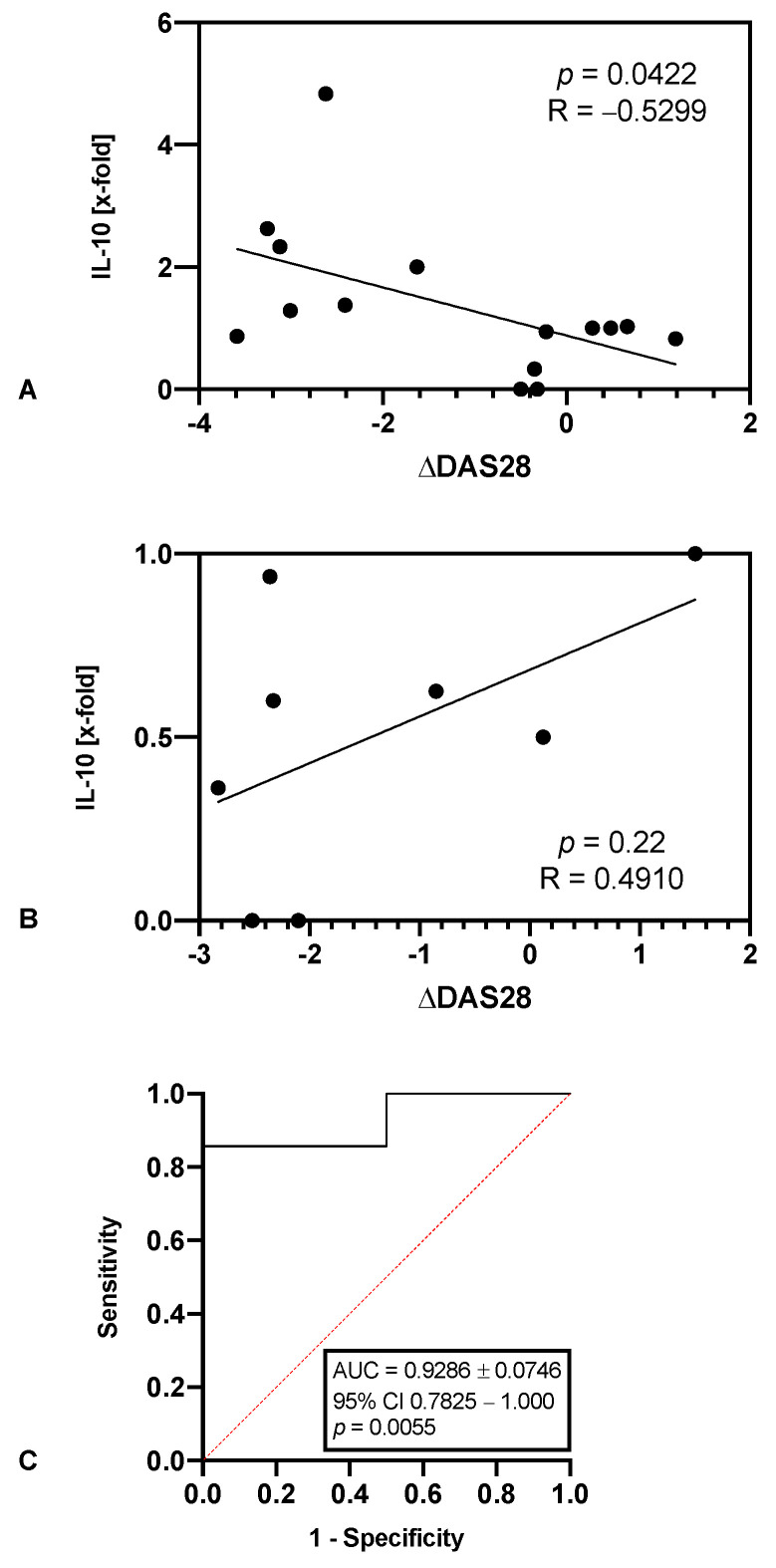
(**A**,**B**) Scatter plot shows the correlation between tmTNF crosslinking-induced IL-10 and the change in DAS28 after initiating treatment with either TNFi (*n* = 15, (**A**)) or JAKi (*n* = 8, (**B**)). Shown is the induced IL-10 production in relation to the human IgG Fc control (x-fold). (**C**) Receiver operating characteristic (ROC) analysis of the value of tmTNF crosslinking-induced IL-10 in predicting TNFi therapy response (*n* = 15). The area under the curve (AUC) is shown with standard error (±SE), 95% confidence interval (CI) and *p* value.

To evaluate the prognostic value of tmTNF crosslinking-induced IL-10 in predicting the therapeutic response, its correlation to a change in DAS28 under TNFi therapy was analysed. In patients treated with TNFi, a significant negative correlation to the change in DAS28 was detected (Figure 3A, R = −0.5299, *p* = 0.0422), while no such link was observed in patients under JAKi therapy (Figure 3B, *p* = 0.22). Receiver operation characteristics (ROC) analysis revealed the high performance of tmTNF/crosslinking-induced IL-10 in predicting a clinical response according to the EULAR criteria (Figure 3C, AUC = 0.9286, 95% Confidence interval 0.7825–1.000, *p* = 0.0055). Using a cut-off > 0.5679-fold, sensitivity and specificity would be 85.7 and 100%, respectively.

## 4. Discussion

An ever-growing armamentarium of therapeutic options available to rheumatologists enables them to treat RA more successful than in the past. Unfortunately, to date, reliable biomarkers to predict the therapeutic response to a specific bDMARD or tsDMARD are missing. Epidemiological parameters can only be helpful to some extent: while male sex is an independent predictor for sustained clinical remission in early RA under TNFi treatment, female sex has been reported to be associated with an increased risk of TNFi failure [14,15]. Since the demand for specific tests aiding therapy decisions is high, different efforts regarding biologic therapy prediction have been made. Most of them though focus on a genomic or proteomic attempt, e.g., [16,17,18,19,20,21].

We report here the development of an immunological whole-blood assay which allows to predict response to TNF inhibition. The results of this pilot study show the tmTNF crosslinking-induced production of IL-10 correlates with both, the initial disease activity and the subsequent response to TNFi. The prognostic value calculated by ROC analysis implies, that the therapeutic response to TNFi can indeed be predicted in a clinically meaningful way. Interestingly, no predictive value for a therapy with JAK inhibitors could be shown. 

This finding emphasizes the TNFi class specific effect which was deduced prior to kit development from our previous experimental in vitro-work on cultured monocytes [10]. Using an in-vitro assay with isolated monocytes, we already demonstrated that tmTNF reverse signalling led to an increased production of IL-10, which was able to predict response to TNFi therapy in RA patients [10]. This principle was translated into a whole-blood assay.

IL-10 is an anti-inflammatory cytokine being produced by different kinds of immune cells including monocytes [22]. Compared to other immune cells, blood monocytes express the highest IL-10 receptor-1 levels and are highly sensitive to IL-10 [23]. In antigen-induced arthritis, IL-10 knockout mice show a more severe disease with increased histological and radiographic joint scores compared to wildtype mice [24]. Similarly, chimeric mice lacking IL-10 producing B cells developed an exacerbated collagen-induced arthritis and demonstrate an increased amount of proinflammatory Th 1 as well as Th 17 cells [25].

In cultured monocytes of RA patients responding to TNFi therapy, we showed increased tmTNF crosslinking-induced IL-10 levels before [10]. Furthermore, the change in DAS28 under TNFi therapy was also associated with baseline IL-10 levels. These findings could be confirmed with the presented whole-blood assay. Additionally, the current results demonstrate a significant reduction of IL-10 approx. 8 weeks after induction of TNF inhibition. Since IL-10 is meant to be an anti-inflammatory cytokine counteracting e.g., TNF and IL-6 production and being induced in inflammation [26,27], the decreasing levels we observed most likely mirror a successful therapy.

## 5. Conclusions

Personalized medicine is the holy grail in today’s therapeutic approach, not only in rheumatology. In this study, we demonstrated the feasibility of a whole-blood immunological assay in predicting response to TNF inhibition in RA patients. This approach could be extraordinarily useful for attending rheumatologist when choosing the right biological therapy for the individual patient. Given the preliminary character of this pilot study, further investigations are clearly needed to validate our whole-blood assay in everyday clinical practise.

## Figures and Tables

**Table 1:** Preparation of the whole blood assay using polyethylene tubes. After coating and drying as outlined above, the tube are sterilized and sodium heparin beads are added to prevent clotting.

Tube	Medium Control	Fc	Etanercept Plate-Bound	Etanercept Plate-Bound+ Solved
Coating protocol	-	970 µL PBS +30 µL Fc fragment (5 mg/mL in PBS)	994 µL PBS +6 µL Etanercept (50 mg/mL)	994 µL PBS +6 µL Etanercept (50 mg/mL) +10 µg Etanercept (pulverized)
Final concentrations in 4 mL whole blood	-	150 µg coated	300 µg coated	300 µg coated + 10 µg solved

Fc—fragment crystallizable; PBS—phosphate buffered saline.

**Table 2:** Patient characteristics and initiated therapy. Given are numbers and % as indicated.

	Result
*Patient characteristics*	
Age in years (mean ± standard deviation)	61.5 ± 12.37
Female, *n* (%)	40 (63.5)
Seropositivity, *n* (%)	61 (96.8)
ACPA positivity, *n* (%)	60 (95.2)
RF positivity, *n* (%)	59 (93.7)
*Initiated DMARD therapy, n (%)*	
TNF inhibitor	15 (23.8)
Adalimumab	3 (20.0)
Etanercept	12 (80.0)
JAK inhibitor	8 (12.7)
Tofacitinib	5 (62.5)
Baricitinib	3 (37.5)
Other/None	40 (63.5)

ACPA—anti-citrullinated protein/peptide antibodies; DMARD—disease-modifying antirheumatic drug; JAK—janus kinase; RF—Rheumatoid factor; TNF—tumor necrosis factor.

## Data Availability

The data underlying this article will be shared on reasonable request to the corresponding author.

## Abbreviations

ACPA	anti-citrullinated protein/peptide antibodies
ACR	American College of Rheumatology
AUC	Area under the curve
bDMARD	biologic disease-modifying antirheumatic drug
CI	Confidence interval
CRP	C-reactive protein
DAS28	Disease activity score 28 joints
DMARD	disease-modifying antirheumatic drug
ELISA	Enzyme-linked immunosorbent assay
EULAR	European League against Rheumatism
Fc	fragment crystallizable
IgG	Immunoglobulin G
IL	interleukin
JAK	Janus kinase
JAKi	JAK inhibitor
PBS	phosphate buffered saline
RF	Rheumatoid factor
ROC	receiver operating characteristic
tmTNF	transmembrane TNF
TNF	tumour necrosis factor
TNFi	TNF inhibitor
tsDMARD	targeted synthetic disease-modifying antirheumatic drug
